# Obesity and Leukemia: Biological Mechanisms, Perspectives, and Challenges

**DOI:** 10.1007/s13679-023-00542-z

**Published:** 2023-12-30

**Authors:** Dimitrios Tsilingiris, Natalia G. Vallianou, Nikolaos Spyrou, Dimitris Kounatidis, Gerasimos Socrates Christodoulatos, Irene Karampela, Maria Dalamaga

**Affiliations:** 1 First Department of Internal Medicine, University Hospital of Alexandroupolis, Democritus University of Thrace, Dragana, 68100 Alexandroupolis, Greece; 2 Department of Internal Medicine, Evangelismos General Hospital, 45-47 Ipsilantou str, 10676 Athens, Greece; 3 Tisch Cancer Institute Icahn School of Medicine at Mount Sinai, 1190 One Gustave L. Levy Place, New York, NY 10029 USA; 4 Department of Microbiology, Sismanogleio General Hospital, 1 Sismanogleiou Str, 15126 Athens, Greece; 5 2nd Department of Critical Care, Medical School, University of Athens, Attikon General University Hospital, 1 Rimini Str, 12462 Athens, Greece; 6 Department of Biological Chemistry, Medical School, National and Kapodistrian University of Athens, 75 Mikras Asias str, 11527 Athens, Greece

**Keywords:** Adipokine, Adiposity, Body mass index, Childhood leukemia, Epidemiology, Leukemia, Obesity

## Abstract

**Purpose of Review:**

To examine the epidemiological data on obesity and leukemia; evaluate the effect of obesity on leukemia outcomes in childhood acute lymphoblastic leukemia (ALL) survivors; assess the potential mechanisms through which obesity may increase the risk of leukemia; and provide the effects of obesity management on leukemia. Preventive (diet, physical exercise, obesity pharmacotherapy, bariatric surgery) measures, repurposing drugs, candidate therapeutic agents targeting oncogenic pathways of obesity and insulin resistance in leukemia as well as challenges of the COVID-19 pandemic are also discussed.

**Recent Findings:**

Obesity has been implicated in the development of 13 cancers, such as breast, endometrial, colon, renal, esophageal cancers, and multiple myeloma. Leukemia is estimated to account for approximately 2.5% and 3.1% of all new cancer incidence and mortality, respectively, while it represents the most frequent cancer in children younger than 5 years. Current evidence indicates that obesity may have an impact on the risk of leukemia. Increased birthweight may be associated with the development of childhood leukemia. Obesity is also associated with worse outcomes and increased mortality in leukemic patients. However, there are several limitations and challenges in meta-analyses and epidemiological studies. In addition, weight gain may occur in a substantial number of childhood ALL survivors while the majority of studies have documented an increased risk of relapse and mortality among patients with childhood ALL and obesity. The main pathophysiological pathways linking obesity to leukemia include bone marrow adipose tissue; hormones such as insulin and the insulin-like growth factor system as well as sex hormones; pro-inflammatory cytokines, such as IL-6 and TNF-α; adipocytokines, such as adiponectin, leptin, resistin, and visfatin; dyslipidemia and lipid signaling; chronic low-grade inflammation and oxidative stress; and other emerging mechanisms.

**Summary:**

Obesity represents a risk factor for leukemia, being among the only known risk factors that could be prevented or modified through weight loss, healthy diet, and physical exercise. Pharmacological interventions, repurposing drugs used for cardiometabolic comorbidities, and bariatric surgery may be recommended for leukemia and obesity-related cancer prevention.

## Introduction

Leukemia constitutes a collection of blood-related malignancies characterized by the transformation of hemopoietic progenitors and the diffuse infiltration of the bone marrow. According to the Fourth Edition of the World Health Organization (WHO) Classification of Tumors of Hematopoietic and Lymphoid Tissues, leukemia can be broadly categorized into myeloid or lymphoid lineages [1]. Based on the course of disease progression (acute or chronic) and the origin of the predominant cell type (lymphoid or myeloid), leukemia is classified into four main types: acute lymphocytic leukemia (ALL), acute myelogenous leukemia (AML), chronic lymphocytic leukemia (CLL), and chronic myelogenous leukemia (CML). Worldwide, based on the GLOBOCAN database, leukemia is estimated to account for approximately 2.5% and 3.1% of all new cancer incidence and mortality in 2020, respectively [2]. Moreover, leukemia represents the most frequent cancer in children younger than 5 years of age accounting for the highest percentage of deaths in this age group [3]. The majority of leukemia cases in childhood are acute, and ALL is the most common type in pediatric populations globally [4].

The etiology of most cases of leukemia has not been elucidated. Leukemia is a multifactorial disease stemming from the interaction of genetic, epigenetic, and environmental factors. Age represents a significant risk factor for cancer including leukemia [5, 6]. With the exception of ALL, leukemia dramatically increases with age, peaking at 80 to 85 years old (y.o.), with a median age at diagnosis between 65 and 72 y.o. [7]. A number of suggested and established risk factors have been implicated, including genetic disorders, certain blood disorders, exposure to ionizing radiation, chemicals such as benzene, and pesticides, infections, cancer treatment with radiotherapy, and/or mutagenic chemotherapy and family history [4, 5, 8–10]. Tobacco smoking and alcohol consumption have also been documented as risk factors in several studies, whereas recent studies have indicated that obesity may contribute to the etiopathogenesis of leukemia [5, 11]. Obesity constitutes a disorder of energy homeostasis which manifests as excessive adipose tissue accumulation [12–14]. As there are no biological markers of overweight and obesity to date, they are diagnosed based on the body mass index (BMI), which is the best and most practical screening test [15–17]. Using the WHO criteria, overweight and obesity are defined as a BMI ≥ 25 and ≥ 30 kg/m^2^ [18]. However, BMI is not the perfect measure, mainly because it does not provide information on the distribution of the adipose tissue (visceral versus subcutaneous), being also insensitive to the ratio of fat to muscle [19, 20]. The global prevalence of obesity has risen dramatically, with more than 670 million adults being obese. It is estimated that, worldwide, almost 39–49% of the global population (around 2.8 to 3.5 billion individuals) has overweight or obesity [21, 22]. Furthermore, childhood obesity represents a global pandemic [23]. Obesity has been associated with a plethora of disorders, including metabolic syndrome, hypertension, type 2 diabetes, cardiovascular disease and risk factors, non-alcoholic fatty liver disease, sleep disorders, polycystic ovary syndrome as well as the severity of COVID-19 and cancer [24–27].

Based on the International Agency for Research on Cancer (IARC) Working Group, there is convincing evidence that excess body weight is associated with an elevated risk for malignancies of at least 13 anatomic sites, including endometrial, esophageal, renal, and pancreatic adenocarcinomas; hepatocellular carcinoma; gastric cardia cancer; meningioma; colorectal, postmenopausal breast, ovarian, gallbladder, and thyroid cancers as well as multiple myeloma [28, 29]. Moreover, there is a strong indication that obesity may be associated with the incidence and mortality of leukemia, particularly AML, CLL, CML, and ALL as well as preleukemic conditions such as myelodysplastic syndromes (MDSs) [11, 30–35].

Whereas obesity may be associated with leukemia based on epidemiologic studies, the biologic rationale and the mechanisms underlying this link remain largely obscure. The goal of this review is to provide an overview of the association between excess body weight and leukemia summarizing important biological mechanisms underpinning this relationship as well as underscoring recent developments on novel insights in pathogenetic mechanisms. Moreover, we give a special emphasis on current epidemiologic evidence and its limitations; the role of bone marrow adiposity in leukemia pathogenesis; the association between obesity and childhood ALL survivors; as well as preventive and therapeutic perspectives and challenges.

## Methodology of the Review

In June 2023, a literature search in the PubMed database was conducted to assess the association between obesity and the risk of leukemia. This search used the following MESH terms: “Obesity” AND “leukemia” AND “risk.” A search of the abovementioned terms yielded a total of 540 results, most of which were published during the past 10 years. Among the 540 studies, 11 were excluded as 3 were written in Polish, 3 in Russian, 2 in Spanish, 2 in Chinese, and 1 in Czech. In addition, 14 studies dealt with cardiovascular (CVD) risk, 14 studies with hyperglycemia and/or insulin resistance, 8 studies referred to nutritional aspects, such as tea or caffeine consumption, 8 studies with other hematologic malignancies (4 with multiple myeloma, 4 with lymphomas), 5 studies with venous thromboembolism events, 5 studies were case reports, and 8 studies dealt with genes and neurological aspects. Therefore, from the 540 studies, 73 studies were excluded, leaving a total of 467 studies.

## Epidemiologic Evidence Linking Obesity to Leukemia

### Evidence from Epidemiologic Studies and Meta-Analyses

Current evidence has suggested a relationship between obesity and leukemia. Indeed, Bhaskaran et al. have documented a significant association between obesity and the risk of leukemia, in their landmark study including 5,240,000 adults, that was published in the Lancet in 2014. In particular, they reported that a 5 kg/m^2^ increase in BMI was almost linearly related to an increased risk of leukemia, among other cancer types [36]. Estimates from the Global Burden of Disease Study, which analyzed data from 1990 to 2017 globally, have reported a significant association between higher BMI and an increased risk of AML [37]. In addition, in 2022, Huang et al. have reported a significant association between obesity and the risk of leukemia [38]. Moreover, in 2023, Ahmed et al. have studied the incidence of various types of cancer among 290,888 participants from the UK BioBank. Totally, 21,973 participants aged 37 to 73 years old, with a median follow-up of approximately 4 years, developed cancer. They concluded that a metabolic profile characterized by an increased BMI in conjunction with increased serum C-reactive protein (CRP) and cystatin C levels may predict an elevated risk of hematologic malignancies in middle age and older people [39]. A broad-scale analysis of cancer-related deaths in the USA between 1982 and 1999 revealed that among other malignancies, a dose-response relationship between BMI and leukemia mortality likely exists, with increasing death rates across overweight, class I, II, and III obesity compared to lean individuals, respectively. These observations were independent of important confounders such as age, nutritional factors, physical activity, tobacco, and alcohol consumption, among others; however, no information regarding different leukemia subtypes was provided [40]. Table 1 depicts major studies associating obesity with an increased risk of leukemia. Overall, there are many studies supporting an association between obesity and an increased risk of all types of leukemia (lymphocytic versus myeloid, acute versus chronic). The presence of obesity is associated with an increased risk of essentially the sum of conditions falling into the spectrum of leukemic disease; this includes CLL and CML, ALL and AML [31], as well as the pre-malignant myelodysplastic syndromes [49]. Although evident in all the aforementioned conditions, the added risk conferred by obesity is likely greater for acute leukemias, especially of lymphoid but also of myeloid origin [31, 50] compared with chronic leukemias. The role of obesity as a risk factor for specific subtypes of AML remains to be fully elucidated; a particularly strong association has been observed for acute promyelocytic leukemia (APL), with an additional 44% risk for each 5 kg/m^2^ increase in BMI [51]. Furthermore, it is unclear whether the putative underlying pathogenetic mechanisms linking obesity to leukemogenesis, which are expanded upon in the following sections, are homogenously implicated in all leukemia subtypes; it is likely that a number of mechanisms are common, whereas others (e.g., perturbations of bone marrow adipose tissue physiology) tend to more selectively partake in the pathogenesis of specific leukemias (in this case, of myeloid origin).

**Table 1:** List of main studies associating obesity with an increased risk of leukemia

Author/year	Study/population	Findings of the study	Comments
Ahmed et al. 2023 [39]	290,888 Participants 21,972 Cases of cancer A population-based study, UK	✓ ↑ Risk for hematologic malignancies [e.g., lymphoid leukemia: HR = 1.83, 95% CI = 1.44 to 2.33] and higher BMI was reported. ✓ Patients’ metabolic profile associated with leukemia risk included ↑ BMI, ↑ serum CRP, and ↑ cystatin C levels.	✓ ↑ BMI, ↑ serum CRP and ↑ cystatin C levels were associated with ↑ risk of leukemia, especially lymphoid leukemia.
Yi et al. 2020 [37]	Estimates from the Global Burden of Disease Study, in 195 countries/territories between 1990 and 2017, 2017	✓ ↑ Risk for AML was reported in association with a higher BMI.	✓ The burden of AML has ↑ during the last years in association with an ↑ in obesity.
Amankwah et al. 2016 [41]	13,921 Cases were included. A meta-analysis study, including 11 studies	✓ ↑ Risk of mortality with ↑ BMI at diagnosis was reported (OS: HR = 1.30, 95% CI = 1.16–1.46 and EFS: HR = 1.46, 95% CI = 1.29–1.64).	✓ ↑ BMI at diagnosis was associated with a poor OS and EFS among children with acute leukemia.
Bhaskaran et al. 2014 [36]	5,240,000 UK adults, among whom 166,955 developed cancer. A population-based cohort study, UK	✓ Each 5 kg/m^2^ increase in BMI was linearly related to the risk of leukemia (1.09, 1.05–1.13; *p* ≤ 0·0001).	✓ ↑ BMI was related to ↑ risk of leukemia, among other cancers.
Jeddi et al. 2010 [42]	39 Patients with APL A study in a Tynisian hospital.	✓ 11 of the 36 patients evaluated for DS (30.5%) developed DS (severe in 7 cases, moderate in 4, and fatal in 3 cases) within a median of 12 days of treatment with ATRA. Six of the 9 (66.6%) patients with BMI ≥ 30 developed DS vs. 5 of 27 (18.5%) with BMI < 30 (*p* = 0.012).	✓ BMI ≥ 30 was a significant predictor of developing DS in APL.
Strom et al. 2009 [43]	253 Cases 270 Controls A hospital based case-control study in Texas, USA	✓ Cases were obese during adulthood, when compared with controls at age 25 [OR = 4.29; 95% CI, 1.63–11.3], at age 40 (OR = 5.12; 95% CI, 1.92–13.6), and at diagnosis (OR = 3.09; 95% CI, 1.56–6.13).	Obesity and weight gain in adulthood are significant risk factors for developing CML.
Wong et al. 2009 [44]	722 Cases of AML 1444 Controls A case-control study in Shanghai, China	✓ An inverse relationship between BMI and overall AML or the sub-category “AML not otherwise categorized,” was reported, whereas a positive association between BMI and the subtype APL was noted.	✓ Categorization by WHO subtypes may not be so significant regarding risk factors for AML.
Larsson et al. 2008 [31]	7,780,338 Participants among whom 17,349 patients with leukemia A meta-analysis of 9 cohort studies	✓ A 5 kg/m^2^ ↑ in BMI was related to a 13% ↑ risk of leukemia (RR, 1.13; 95% CI, 1.07–1.19). ✓ In a meta-analysis of 4 studies, the RRs related to obesity were 1.25 (95% CI, 1.11–1.41) for CLL, 1.65 (95% CI, 1.16–2.35) for ALL, 1.52 (95% CI, 1.19–1.95) for AML and 1.26 (95% CI, 1.09–1.46) for CML.	✓ This meta-analysis supports that ↑ BMI is associated with ↑ risk of leukemia, either acute or chronic forms.
Chiu et al. 2006 [45]	35,420 Participants A cohort study in Chicago, USA	✓ For women, there was a trend for ↑ mortality from leukemia with ↑ BMI (HR, 2.47; 95% CI, 0.96–6.36; *p* = 0.02).	✓ A trend for an association between ↑ BMI and ↑ mortality from leukemia was noted only in women.
Kasim et al. 2005 [46]	1068 Cases 5039 Controls A population-based cohort study in Canada	✓ The authors reported a relationship between the highest BMI for AML, CML, and CLL, with a dose-response association.	✓ The highest BMI was associated with ↑ risk for AML, CML and CLL.
Ross et al. 2004 [47]	Over 40,000 Iowa women, by questionnaire. 200 Women developed leukemia: 74 AML and 88 CML. During follow-up. Minneapolis, USA	✓ The risk of AML was ↑ among women, who had reported an ↑ BMI (RR for overweight, 1.9; 95% CI, 1.0–3.4; RR for obese, 2.4; 95% CI, 1.3–4.5; *p* = 0.006), when compared with women of normal BMI.	✓ A trend between ↑ BMI and ↑ risk for AML and CLL was reported.
Estey et al. 1997 [48]	1245 Patients with AML whom 120 had APL. A hospital-based cohort study in Texas, USA	✓ ↑ BMI has a positive relationship with diagnosis of APL (*p* = 0.0003).	✓ The authors reported a strong association between ↑ BMI and ↑ risk for APL.

*ALL* acute lymphocytic leukemia, *AML* acute myeloid leukemia, *APL* acute promyelocytic leukemia, *ATRA* ALL trans retinoic acid, *BMI* body mass index, *CI* confidence intervals, *CLL* chronic lymphocytic leukemia, *CML* chronic myeloid leukemia, *DS* differentiation syndrome, *EFS* event-free survival, *HR* hazards ratio, *OS* overall survival, *RR* relative risk, *vs* versus

### Birth Weight and Childhood Leukemia

There is a growing body of evidence suggesting that an increased birthweight (BW), usually defined as ≥ 4000 g, may be associated with the development of childhood leukemia. This association may be attributed to increased levels of growth hormone (GH) and insulin-like growth factors (IGF) in infants, who have later developed leukemia [52, 53]. As GH and IGF are also related with an increased stature, it has been postulated that increased height at diagnosis of ALL may be observed among children with ALL [54]. However, although Huang et al. have reported this positive association between height at diagnosis and ALL, later studies have questioned this relationship [55]. Schraw et al. have attributed these apparently different findings to the selection of distinct populations studied in diverse clinical settings and with different reference data used [55]. Nevertheless, even though height at diagnosis of childhood ALL may not be a consistent finding, BW seems to be a risk factor for developing childhood ALL [56]. In addition, newborns larger for gestational age were documented to be at a higher risk for childhood ALL [57]. Table 2 depicts main studies associating elevated BW with an increased likelihood of childhood leukemia. Interestingly, a body of epidemiological studies, including a recent meta-analysis, have documented a relationship between maternal obesity and leukemia in the offspring; nevertheless, the pathogenetic mechanisms underlying this relation are unclear [61, 62].

**Table 2:** List of main studies associating an increased birthweight (BW) with an increased risk for childhood leukemia

Author/year	Study population	Findings of the study	Remarks
Stacy et al. 2019 [57]	1,827,875 Infants with 747 children being diagnosed with leukemia before the age of 14 y.o. A prospective cohort analysis.	✓ Children born to mothers with a BMI ≥ 40 had a 57% (95% CI: 12, 120) higher risk for childhood leukemia. ✓ Newborn size of ≥ 30% higher than expected related to a 1.8-fold HR for leukemia, when compared to those with the expected size.	✓ Maternal obesity and increased newborn size may be involved in an ↑ risk for developing childhood leukemia. ✓ Increased insulin and IGFs may underlie these findings.
Jimenez-Hernadez et al. 2018 [58••]	1455 Children with leukemia. 1455 controls. 0–18 y.o. A case-control study	✓ A significant relationship between ALL and child’s birthweight ≥ 2500 g was reported (aOR 2.06; 95% CI: 1.59, 2.66). ✓ This association was observed in those with birthweight ≥ 3500 g as well (aOR 1.19; 95% CI: 1.00, 1.41)	✓ BW ≥ 3500 g was related to ALL and AML. ✓ There was a relationship between ↑ BW and ↑ risk of acute leukemia.
Tran et al. 2017 [59]	124 Children with leukemia 822 Controls A case-control study	✓ ORs of leukemia risk for children with low and high BWs were 0.8 (95% CI: 0.2, 3.0) and 1.4 (95% CI: 0.7, 2.6), respectively.	✓ No statistically significant relationship between BW and childhood leukemia was noted. ✓ However, this study was performed mainly to estimate the risk for CNS tumors among different BW.
Sprehe et al. 2010 [56]	2254 Children with cancer aged < 5 y.o. at cancer diagnosis 11,734 Controls, matched for age A total of 13,988 children A retrospective chart review	✓ Children with BW LGA at birth had a 1.66 (95% CI 1.32–2.10) higher odds of ALL, compared to children with BW AGA. ✓ Children with a BW ≥ 4000 g had a 1.5 (95% CI 1.18–1.89) higher odds for ALL, compared to children who had BW > 2500 and < 4000 g. ✓ ORs were similar for LGA children who were < 4000 g and LGA children who were ≥ 4000 g (OR: 1.5, 95% CI 0.97–2.5 and OR: 1.67, 95% CI 1.29–2.16, respectively)	BW, especially when corrected for gestational age was a better predictive factor of BW alone for the development of ALL.
Milne et al. 2009 [52]	347 Children with cancer aged 0–14 y.o. 762 Controls aged 0–14 y.o. A case-control study	✓ Risk of ALL was related to proportion of optimal BW; the OR for a 1 standard deviation ↑ in proportion of optimal BW was 1.18 (95% confidence interval: 1.04, 1.35) after adjustment for matching variables and various confounders.	✓ Accelerated growth at birth, even more than BW per se, was related to ↑ risk for ALL. ✓ A potential implication for IGFs may underlie the abovementioned findings.
Caughey et al. 2009 [53]	16,501 Children with leukemia, aged ≤ 30 y.o. In particular: 10,974 Children with ALL. 1832 Children with AML. A meta-analysis of 32 studies.	✓ OR for the relationship between high BW and ALL and AML were 1.23 (95% CI: 1.15, 1.32) and 1.40 (95% CI: 1.11, 1.76), respectively, when compared to normal BW. ✓ ↑ BW was not related to overall and ALL leukemia, but only to AML (OR = 1.50; 95% CI: 1.05, 2.13).	✓ This meta-analysis reported an ↑ risk for overall cases of leukemia as well as ALL with regards to ↑ BW.
Hjalgrim et al. 2003 [60]	10,282 Children with leukemia. A meta-analysis of 18 epidemiological studies	✓ Children with BW ≥ 4000 g have an ↑ risk of ALL, when compared with children with ↓ BW (OR = 1.26, 95% CI: 1.17, 1.37)	A dose-response-like effect for the association between BW and ALL.

*AGA* appropriate for gestational age, *BMI* body mass index, *BW* birth weight, *CNS* central nervous system, *HR* hazard ratio, *IGFs* insulin growth factors, *LGA* large for gestational age, *OR* odds ratio, *y.o.* years old

### Limitations of Epidemiologic Studies and Meta-Analyses

Notwithstanding that most studies have depicted a relationship between obesity and the risk of leukemia, there are several limitations in meta-analyses and epidemiological studies. First, when interpreting the included meta-analyses, the inherent limitations of the original studies should be taken into account. For example, the main indicator of obesity, i.e., BMI measurement, was inconsistent with variations from WHO-specified criteria while self-reported questionnaires were commonly used instead of objective measures, which may have influenced the accuracy of the results. In addition, BMI is a practical measure of obesity, but has some inherent drawbacks. It has been demonstrated that BMI does not reflect the body fat distribution and the subsequent CVD risks associated with adiposity [19, 20, 57, 63]. Moreover, other studies have shown decreased response rate amid control participants, small number of included studies, and limited statistical power. Many studies were retrospective which are prone to selection bias in comparison to cohort studies. Other investigations have shown heterogeneous results, while publication bias must be taken into account in systematic reviews [64]. Therefore, limitations exist when comparing different studies; nevertheless, the general tendency of an association between obesity and leukemia risk should not be overlooked.

## Obesity and Childhood ALL Survivors

### Weight Gain Among Survivors of Childhood

Despite the rise in overweight/obesity rates in childhood as well as in adulthood, most children with ALL have normal weight at diagnosis of ALL. However, during or after treatment of childhood ALL, substantial increases in weight have been documented. More specifically, as many as 50% of childhood ALL survivors have increased body weight and this weight gain has been attributed to multiple factors [65].

The Childhood Cancer Survivor Study (CCSS) was conducted by 26 medical centers in the USA and Canada enrolling more than 14,000 cancer survivors, who were diagnosed between 1970 and 1986 [66]. The CCSS has reported a 20% increase in obesity among males and a 50% increase among female survivors [66]. In addition, in a meta-analysis among 9223 pediatric ALL survivors, Zhang et al. have concluded that obesity was much more prevalent in ALL survivors, when compared to the reference group [67]. More specifically, the majority of studies had enrolled survivors who were off treatment for less than 5 years, whereas only a small number of studies included survivors who were off treatment for more than 10 years. In particular, among patients who were off treatment for at least 10 years, prevalence of obesity was between 34% and 64% [67]. It is noteworthy that subgroup analysis has demonstrated obesity to be more prevalent regardless of their age at onset of ALL, their gender, or the previously administered cranial radiation therapy (CRT) or not [67]. Very recently, Richard et al. have reported the results from the CCSS and the St. Jude Lifetime Cohort (SJLIFE) studies regarding genetic variants in adult survivors of childhood ALL [68•]. By using Genome-Wide Association Study (GWAS), they have documented that more than 700 loci are responsible for 6.2% of the genetic variation of BMI in adult survivors of childhood ALL. They have confirmed that ALL survivors have approximately the same genetic heritability as the general population regarding BMI. However, CRT may modify BMI-associated loci among adult survivors of childhood ALL [68•]. Furthermore, Green et al. have shown that CRT, physical inactivity, and the use of certain anti-depressant medication are correlated with increased BMI among pediatric ALL survivors in adulthood [69]. In an Epigenome-Wide Association Study (EWAS), Wahl et al. have documented that variations in BMI, as a marker of adiposity, are correlated with changes in DNA methylation at cytosine-guanine sites [70]. Indeed, Lupo et al. have studied 96 pediatric ALL survivors and have shown that 39 loci were related to obesity among the pediatric ALL survivors, who received only chemotherapy and not CRT [71••]. Therefore, with the use of WGAS and EWAS, researchers are now able to confirm that different molecular pathways are involved in the development of obesity among pediatric ALL adult survivors, who received only chemotherapy or only CRT.

Overall, weight gain may occur in a substantial number of childhood ALL survivors via different molecular pathways. Chemotherapy, CRT, and the administration of corticosteroids may be implicated in the development of obesity among pediatric ALL survivors.

### Relationship Between Excess Body Weight and Risk of Relapse and Mortality

There is a growing body of evidence supporting the notion that adiposity is associated with a decreased efficacy of ALL treatment [72]. This notion is based upon the fact that lymphoblasts have been documented to migrate into the adipose tissue [73]. Notably, lymphoblasts in the adipose tissue are protected from degradation, while adipose-derived stromal/stem cells secrete factors, which have been implicated in the proliferation of lymphoblasts [74]. Thus, the combination of proliferation and protection of lymphoblasts in the excess adipose tissue accounts for the increased risk of mortality in ALL patients, which has been attributed to obesity [74, 75]. More specifically, adipocytes secrete lipids and amino acids, which support the growth and proliferation of leukemia initiating cells (LICs) [76, 77]. In addition, Lee et al. have reported that adipocytes may induce the expression of Galectin-9 (GAL-9) on the surface of B-ALL cells in humans [78]. They have confirmed the enhanced expression of GAL-9 on B-ALL cells among pediatric patients with obesity, when compared to lean patients with pediatric B-ALL [78]. They have also documented that in relapse, higher gene expression of GAL-9 has been correlated to poorer outcomes [78]. Therefore, increased GAL-9 expression may exert “protective effects” on B-ALL cells [78]. Moreover, apart from the protective effects of adipocytes on LICs, obesity could induce alterations in the pharmacokinetics of various chemotherapeutic agents through multiple mechanisms. For example, the accumulation of lipid-soluble chemotherapeutics and the enhancement in the secretion of water-soluble drugs may lead to changes in the metabolism of chemotherapeutic agents [79]. Besides, further metabolism of chemotherapeutic agents, such as doxorubicin and daunorubicin via reductases, may also contribute to changes in the efficacy of the abovementioned anthracyclines [80].

Higher adiposity, chiefly indexed by increased BMI, has been associated with adverse leukemia outcomes in adults, although the overall findings are controversial, and the effects are likely lineage specific. In a recent study among adolescent and adult younger ALL patients aged younger than 50 years, elevated BMI was independently associated with increased treatment toxicity (mainly hepatotoxicity and hyperglycemia), higher relapse-free mortality, and shorter overall survival, effects which were more pronounced among the higher age groups [81•]. An earlier report demonstrated an increased 5-year mortality among adults with ALL and obesity (HR 1.60, 95% CI 1.03–2.50, *p* = 0.035). This was exclusively driven by the increased mortality in the subset of patients with ALL of T-lymphocyte lineage (HR 5.42, 95% CI 1.84–15.98, *p* < 0.001), while no impact of BMI was observed on patients with B-ALL [82]. On the other hand, among 1974 newly diagnosed cases of AML in adult patients, obesity was associated with better rates of complete remission and lower incidence of treatment-resistance AML, without any noted effects on survival [83]. In contrast, another study reported worse overall survival among patients with AML and obesity (aHR 0.6, *p* = 0.03), a finding which was independent of comorbidity burden, age, cytogenetic features of AML, or treatment intensity [84]. The discrepant findings regarding the impact of obesity on AML prognosis may at least partially be attributable to the differential effects according to AML subtypes. Based on a relevant meta-analysis, obesity seems to adversely affect prognosis particularly in APL [85].

Table 3 depicts major studies examining the association between obesity and the risk of relapse or mortality among patients with pediatric ALL. The majority of studies have documented an increased risk of relapse as well as increased mortality rates among patients with childhood ALL and obesity [75, 89, 91]. However, a minority of studies have not confirmed this correlation [86]. The discrepancy of results may be due to differences in ethnic groups. Of note, Mexican children with ALL possess *ETV6-RUNX1* in only about 6%, a gene rearrangement, which seems to be related to a better outcome, whereas in developed countries, this gene rearrangement is present in 22% approximately [92]. Apart from differences in ethnic groups, different sample sizes as well as variations in confounding factors, adjustments in statistical analyses and median follow-up times may all be associated with discrepancies of results. Therefore, further large-scale studies are needed to confirm the relationship between increased risk of relapse and mortality among obese patients with childhood ALL, when compared to normal weight patients with ALL.

**Table 3:** List of meta-analyses and epidemiologic studies associating risk of relapse and mortality in pediatric ALL with overweight/obesity

Research/year	Population/type of study	Main findings	Remarks
Baillargeon et al. 2006 [86]	322 Pediatric patients with B-precursor ALL, aged 2–18 y.o. Retrospective cohort study	Obesity at diagnosis was not related to ↓ OS (HR: 1.40, 95% CI: 0.69–2.87) or ↓ EFS (HR: 1.08, 95% CI: 0.65–1.82) in the overall study or in either of the age subgroups: 2–9 y.o. and 10–18 y.o.	✓ Patients were mainly of Hispanic white origin. ✓ No association between obesity and OS/EFS was noted.
Ethier et al. 2012 [87]	238 Patients with ALL aged 2–18 y.o. Retrospective chart review	Patients with ↑ BMI had ↓ 5-year EFS (62.2 ± 12.1% vs. 83.6 ± 2.6%; *p* = 0.02) and OS (80.7 ± 8.7% vs. 92. ± 1.9%; *p* = 0.005).	✓ Obese patients with childhood ALL have ↓ OS.
Aldhafiri et al. 2014 [88]	1033 Patients with ALL aged 2–18 y.o. National cohort study (UK)	No evidence that weight at diagnosis was associated with risk of relapse: log-rank test (*p* = 0.90) with overweight and obesity as the exposure (*n* = 917); individual (*p* = 0.42) and stepwise (*p* = 0.96) proportional hazard models.	✓ Overweight/obesity does not change the prognosis of childhood ALL.
Orgel et al. 2016 [89]	8680 Patients with ALL aged 0–21 y.o. Meta-analysis from 11 studies	↓ EFS in patients with an ↑ BMI (RR: 1.35; 95% CI: 1.20-1.51) compared to those with ↓ BMI. There was a statistically non-significant trend towards ↑ risk of relapse (RR: 1.17; 95% CI: 0.99-1.38) in patients with ↑ BMI.	✓ An ↑ BMI at diagnosis was related to ↑ mortality in ALL patients (RR: 1.31; 95% CI: 1.09-1.58).
Amankwah et al. 2016 [41]	13,921 Patients aged < 21 y.o. Meta-analysis from 11 studies	↑ Risk of mortality with ↑ BMI (OS: HR: 1.30, 95% CI: 1.16–1.46 and EFS: HR: 1.46, 95% CI: 1.29–1.64).	✓ Targeting obesity in pediatric ALL patients may improve OS.
Saenz et al. 2018 [90]	181 Pediatric leukemia patients aged 2–17 y.o. Retrospective cohort study and meta-analysis	The present study did not show a significant relationship between obesity and mortality from ALL. Pooled analysis showed a relationship between overweight/obesity and ↑↑ mortality in ALL (HR: 1.39, 95% CI: 1.16–1.46). In children > 10 y.o., a relationship between obesity and relapse was observed.	Small sample size ✓ ↑ Risk of mortality among children with overweight/obesity in the meta-analysis.
Nunez-Enriquez et al. 2019 [75]	1070 Children with ALL aged < 15 y.o. Multicenter cohort study	Overweight/obesity at diagnosis was a predictive factor of early mortality (WHO: HR: 1.4, 95%, CI: 1.0–2.0; CDC: HR: 1.6, 95% CI: 1.1–2.3). No relationship between overweight (WHO: HR: 1.5, 95% CI: 0.9–2.5; CDC: HR: 1.0; 95% CI: 0.6–1.6) and obesity (WHO: HR: 1.5, 95% CI: 0.7–3.2; CDC: HR: 1.4; 95% CI: 0.9–2.3) with early relapse was noted.	✓ Overweight and obesity either according to WHO or CDC criteria were associated with early mortality in childhood ALL. ✓ However, overweight/obesity was not related to early relapses.

*ALL* acute lymphoblastic leukemia, *BMI* body mass index, *CDC* Centers for Disease Control and Prevention, *CI* confidence intervals, *EFS* event-free survival, *HR* hazard ratio, *OR* odds ratio, *OS* overall survival, *PFS* progression-free survival, *RCT* randomized controlled trials, *RR* relative risk, *WHO* World Health Organization, *y.o.* years old

## Biological Mechanisms Associating Obesity with Leukemia

**Fig. 1:**
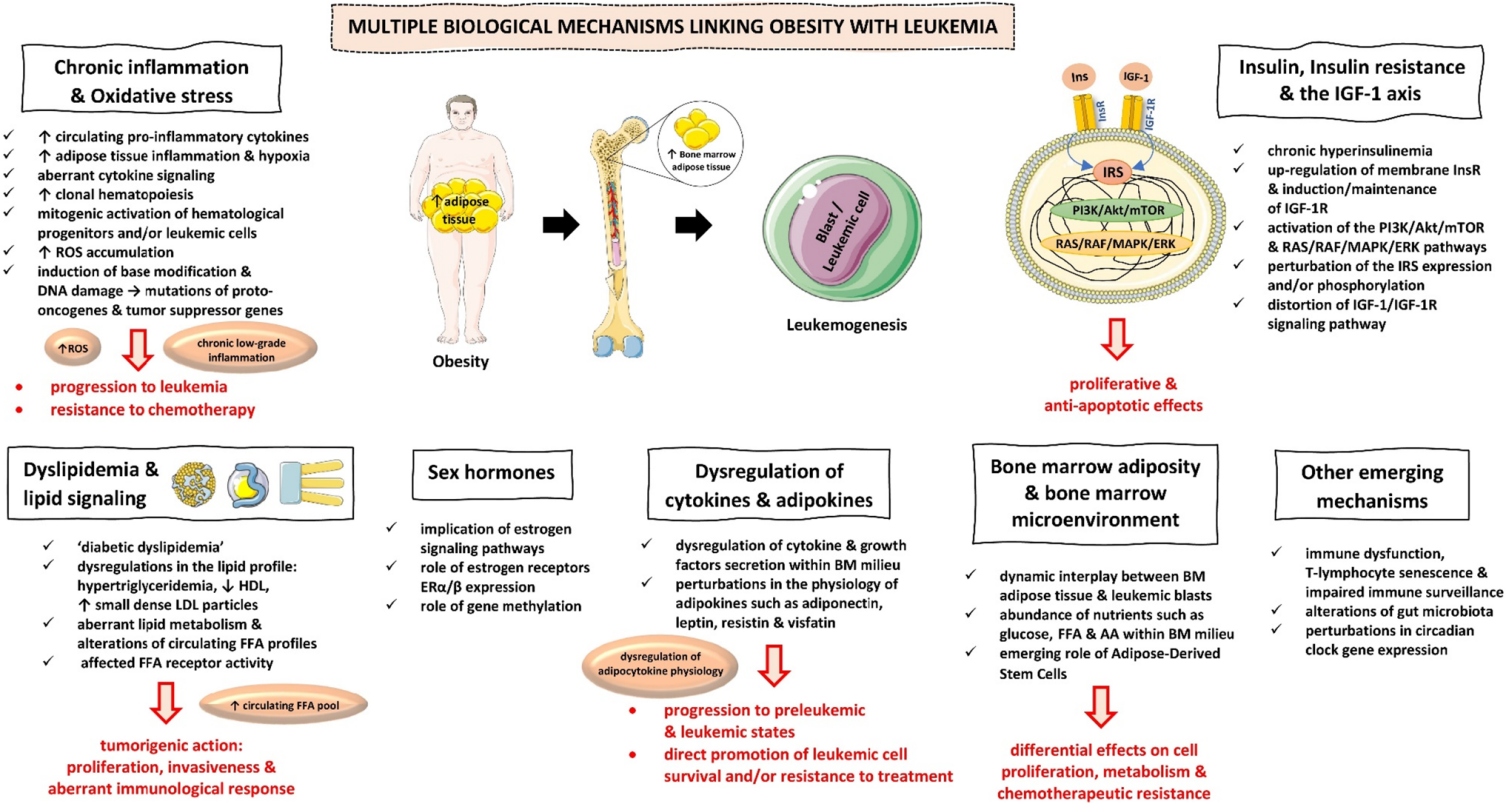
The pathophysiological mechanisms linking obesity to leukemia. AAs, aminoacids; BM, bone marrow; ER, estrogen receptor; FFAs, free fatty acids; HDL, high-density lipoprotein; IGF, insulin-like growth factor; Ins, insulin; InsR, insulin receptor; IRS, insulin receptor substrates; LDL, low-density lipoprotein; ROS, reactive oxygen species. All images are originated from the free medical site http://smart.servier.com/ (accessed on August 7, 2023) by Servier licensed under a Creative Commons Attribution 3.0 Unported License

Aside from the main properties of the adipose tissue which encompass energy storage and thermal insulation, the adipose tissue is the largest endocrine organ that secretes a plethora of bioactive polypeptides, called “adipokines” or “adipocytokines” [16]. White, beige/brite, brown, and pink fat tissues represent the main types of adipose tissue, while all four types of adipocytes have endocrine functions [93–95]. Adipocytes are also present in the bone marrow and marrow adipose tissue (BMAT) representing about 10% of the human organism’s total fat tissue mass [96]. Obesity may lead to an enlargement of the BMAT size [97]. Interestingly, in the bone marrow milieu, there exists a network among leukemic blasts, hematopoietic stem cells (HSCs), adipocytes, pre-adipocytes, and other cells, such as osteoblasts, osteoclasts, and osteocytes via signaling molecules [98, 99]. The pathophysiological mechanisms linking obesity to leukemia are presented in Fig. 1. Although the role of excess body weight in leukemia etiopathogenesis is not fully elucidated, and the main pathways linking obesity adiposopathy to leukemia are complicated and comprise BMAT; hormones including insulin and the insulin-like growth factor system as well as sex hormones; pro-inflammatory cytokines and growth factors, such as IL-6 and TNF-α; adipocytokines, such as adiponectin, leptin, resistin, and visfatin; dyslipidemia and lipid signaling; chronic low-grade inflammation and oxidative stress; and other emerging mechanisms.

### Insulin, Insulin Resistance, and the IGF-1 Axis

Insulin resistance represents a pathological state defined as a condition of lower insulin-targeting tissue responsiveness to insulin levels [100–102]. Obesity constitutes a chronic hyperinsulinemic state, and when insulin secretion can no longer compensate for insulin resistance, metabolic syndrome and type 2 diabetes mellitus (DM) may develop [27, 35, 103]. Chronic hyperinsulinemia is related with an elevated risk of several obesity-related cancers, such as breast, endometrial, ovarian, and prostate cancers [104–108].

Multiple levels of the signaling pathways of insulin and IGF-1 are of capital importance in the pathogenesis of leukemia. Under normal conditions, healthy cells, including lymphocytes, exhibit low levels of surface insulin receptor (InsR) expression, due to the degradation of InsR following insulin binding [109]. The upregulation of membrane InsR has been recognized as a tumorigenesis-promoting mechanism in certain solid malignancies and CLL [110], although corresponding evidence regarding acute leukemias is lacking [111]. Likewise, IGF-1 receptor in T-ALL is maintained high by Notch signaling [112], and is induced in B-ALL by HoxA9 overexpression and occasionally in AML, thus promoting leukemogenesis [113, 114].

Following insulin or IGF-1 binding to their receptors, further signal transduction involves the phosphorylation of the insulin receptor substrates (IRS) and the subsequent activation of the phosphoinositide 3-kinase-Akt-mammalian target of rapamycin (PI3K/Akt/mTOR) pathway [115]. Furthermore, cytokine as well as steroid and other hormone receptors and integrins also utilize IRS phosphorylation in order to regulate cellular metabolism, growth, differentiation, or proliferation [116]. This particular role of IRS as effectors of versatile extracellular signals, which include not only insulin and IGF-1 but also interleukins (ILs) and other cytokines elevated in the systemic environment of chronic low-grade inflammation observed in obesity and insulin resistance, renders them and their related pathways key intersection points in the pathogenesis of leukemic disease in obesity. Intracellular signaling involving the IRS1/2 is implicated in normal hematopoiesis, and perturbation of the IRS expression and/or its phosphorylation status have been implicated in leukemogenesis [117]. The IRS1/2 signaling interrelates with the BCR/ABL [118] or JAK2 pathways in chronic myeloproliferative disorders [119]. Activating mutations of the IRS-2 have been implicated in the pathogenesis of chronic myeloid leukemia refractory to tyrosine kinase inhibitor treatment [120]. The knockdown of IRS1/2 or their targeting by overexpression of miR-570 suppresses glucose metabolism, inhibits proliferation, and induces apoptosis of CML cells in vitro [121]*.* Likewise, the IRS-2 overexpression is observed in patients with non-CML chronic myeloproliferative disorders; the silencing of IRS-2 reduced cell viability and increased apoptosis in cells harboring the pathogenetic JAK2V617F mutation and enhanced the effects of JAK1/2 inhibitor ruxolitinib [122]. Mutations of IRS2 have also been identified in chronic myeloproliferative neoplasms which do not exhibit the most common mutation in JAK2, MPL, and CALR genes [123]. IRS1 is also overexpressed in ALL cells compared with normal hematopoietic cells, despite similar levels of IGF-1R expression [124]. Increased expression of IRS1 in adult BCR/ABL-positive B-ALL is associated with lower survival independently of age and leukocyte count at diagnosis [125]. Similarly, in vitro treatment of pre-B-ALL cells with BCR-ABL inhibitor GZD824 downregulates IRS-1 and the subsequent activation of the PI3K/AKT pathway, inducing cell cycle arrest and promoting apoptosis [126].

The downstream proliferative and anti-apoptotic effects of insulin and IGF-1 signaling are mediated by the activation of the PI3K/Akt/mTOR and RAS/RAF/MAPK/ERK pathways [127, 128]. Subsequently, targeting components of these pathways via newly developed agents or repurposed drugs from the obesity/type 2 diabetes armamentarium constitutes an attractive putative strategy in the treatment of leukemic disease [113, 114, 129–131] (see also “Candidate Therapeutic Agents Targeting Oncogenic Pathways of Obesity and Insulin Resistance in Leukemia” section). Moreover, in ALL, hyperglycemic patients undergoing induction have shorter durations of remission and median survival compared to normoglycemic patients [132]. Mechanistic studies have suggested high levels of insulin/insulin signaling as the underpinning mechanism of this finding [133].

Distorted IGF-1/IGF-1R signaling has been linked to the development of aggressive and/or refractory leukemia [114, 134–136]. In the context of pediatric AML, dysregulation of this pathway has been associated with treatment failure and decrease relapse-free survival in both the setting of induction chemotherapy and HSC transplantation [134–136]. A study in 30 AML patients showed that elevated serum levels of the insulin-like growth factor binding protein (IGFBP) family were associated with worse progression-free survival and overall survival, suggesting that outcomes in myeloid leukemias may be influenced by IGFBPs, probably mediated through the alteration of IGF-1R activation [136]. In a broader context, it has been suggested that AML cells native to the adipose tissue of affected patients may induce the production of IGFBP1 by adipocytes leading to a state of systemic insulin resistance and directly act as a mitogenic signal on CML cells through the Erk signaling pathway [137].

In summary, alterations of InsR and IGF-1r signaling are observed in various leukemia types, which ultimately contribute to the leukemic cell proliferation, evasion of apoptosis, and/or resistance to treatment. Furthermore, components of these pathways may serve as potential targets for anti-leukemic treatment.

### Dyslipidemia and Lipid Signaling

Obesity is accompanied by atherogenic dyslipidemia, which is characterized by quantitative and qualitative changes of plasma lipoproteins [138–140]. The major dysregulations in the lipid profile comprise hypertriglyceridemia, reduced high-density lipoprotein (HDL) cholesterol level, and elevated small dense low-density lipoprotein (LDL) particles [138]. Dyslipidemia has also been linked to high cancer incidence and mortality in solid tumors [141]. Early findings have suggested that patients with hematologic malignancies display lipid profile abnormalities that are proportional to the tumor burden [142]. The Metabolic Syndrome and Cancer Project that focused on 578,000 adults identified that total cholesterol and triglyceride levels are inversely correlated with the incidence of myeloid neoplasms [143]. Since then, and based on this premise, statin therapy has been used in efforts to increase chemotherapy efficacy in AML with promising results [144, 145]. However, these studies were phase I/II trials, and further investigation in the randomized setting is warranted.

Lipid signaling is one potential pathway through which obesity may promote cancer. Obese people exhibit higher concentrations of circulating free fatty acids (FFAs), prominently as a manifestation of adipocyte insulin resistance which results from the failure of circulating insulin to suppress lipolysis [146], leading further to the aggravation of insulin resistance in peripheral tissues [147]. On the other hand, the presence of obesity and related conditions is associated not only with elevated concentrations but also qualitative shifts in the circulating FFA pool [148, 149], which may differentially affect the FFA receptor activity [150].

Alterations of circulating FFA profiles are also observed in acute leukemias and pre-leukemic conditions such as myelodysplastic syndromes and aplastic anemia [151]. Furthermore, adipocytes exhibit a release of FFAs in the presence of ALL cells, which are in turn stored intracellularly in leukemic cells for on-demand energy production or act as building blocks for the production of other macromolecules [152]. Leukemic cells in relapsed AML exhibit aberrant lipid metabolism, with increases of highly unsaturated and long-chain fatty acids, sphingomyelins, and triglycerides, among others [153].

The monoacylglycerol lipase pathway may promote the upregulation of FFAs in cancer cells [154]. These fatty acids could be turned into tumorigenic signaling lipids, through the fatty acid synthase [155]. These lipid signaling molecules include lysophosphatidic acid, prostaglandins, sphingosine-1-phosphate (S1P), platelet activating factor, and phosphoinositides, which may promote tumorigenic pathways including proliferation, invasiveness, and aberrant immunological response [156]. S1P is of particular interest for leukemia as it stimulates the growth and survival of leukemia and lymphoma cells through the NF-kappa B pathway [157].

Elevated circulating triglyceride and lower HDL-cholesterol levels have been consistently observed in ALL and AML [158–162]. Accordingly, overall survival in AML patients has been shown to be independently associated with elevated triglyceride and reduced HDL levels before treatment initiation [163]. Of note, this pattern is consistent with the so-called diabetic dyslipidemia, which likely emerges as a result of insulin resistance and increased production of large buoyant VLDL1 particles by the liver [164, 165]. Hence, it would seem possible that the observed associations may be mediated by the presence of insulin resistance and not as a direct consequence of altered lipoprotein levels. In this regard, a retrospective study among 712 newly diagnosed AML cases (319 acute promyelocytic and 393 non-promyelocytic) has shown the presence of elevated triglycerides and lower HDL as risk factors for higher initial leukocyte counts and early death in APL. Furthermore, it highlighted the role of the increased peroxisome proliferator-activated receptor alpha (PPARα) expression as a common denominator for increased triglycerides and leukemic cell proliferation [166].

### Sex Hormones

Increased adipose tissue affects sex hormone physiology in both genders [167]. With excess body weight, the levels of testosterone diminish in men with obesity, whereas obese women, especially those with an abdominal phenotype, may present a state known as functional hyperandrogenism [168, 169]. Epidemiological studies have found that the incidence of hematological malignancies varies depending on the sex. Since males are about twice as likely to be diagnosed with ALL or CLL and other lymphomas, it has been hypothesized that estrogen may act as a preventative factor in the onset of these neoplasms [170, 171]. Estrogen signaling pathways have recently been implicated in normal hematopoiesis [172].

Estrogen receptor alpha (ERα) induces cells to growth and is expressed throughout the body, including the hematopoietic tissue [173]. On the other hand, estrogen receptor beta (ERβ) exerts anti-proliferative effects and is expressed in the bone marrow, lung, colon, breast, and prostate. In the context of blood cancers, researchers found that the ERα CpG island is abnormally methylated in a big proportion of all malignant neoplasms and ~ 90% of samples of AML patients [174]. This methylation pattern is mostly observed in normal karyotype AML and leads to the downregulation of ERα expression. However, there is conflicting evidence about the role of gene methylation and long-term patient outcomes. About a third of the genes commonly associated with AML biology have been shown to be upregulated by ERα [175]. It is very difficult to establish causality of methylation and carcinogenesis as the genetic alterations in AML often affect the epigenetic landscape of the blasts.

In AML, ERβ is more highly expressed than ERα in some AML patient gene sets [176, 177]. High ERβ/ERα ratios may contribute to the potential role of ERβ signaling against leukemia [178]. Nevertheless, data on the impact of ERβ signaling are limited while its role is not clear.

### Chronic Inflammation and Oxidative Stress

Chronic systemic low-grade inflammation is a hallmark feature of obesity and insulin resistance. A multitude of mechanisms contribute to an increase of circulating levels of pro-inflammatory cytokines in obesity, in conjunction with adipose tissue inflammation, dysfunction and hypoxia, and deteriorating insulin resistance [179] (see also “Dysregulation of Cytokines and Adipokines” section). Concurrently, a spectrum of pro-inflammatory cytokine levels overlapping with those elevated in obesity appear increased ALL and AML [180, 181], while aberrant cytokine signaling is a consistent pathogenetic feature of cell proliferation, survival, and resistance to chemotherapy in leukemia [182]. Hence, a plausible hypothesis could implicate the mitogenic activation of hematological progenitors and/or leukemic cells by the chronically elevated cytokines in obesity as a putative link between increased adiposity and leukemogenesis. According to a recent study, central obesity indexed by an elevated waist-to-hip ratio is associated with the presence of clonal hematopoiesis of indeterminate potential [183], a condition associated with a yearly risk of 0.5–1% for leukemia [184]. Mechanistic data from the same study has revealed that this relationship is likely mediated by the excessive inflammatory environment accompanying increased adiposity [183].

In the context of AML, chronic inflammation is a feature of MDS progression to AML [185]. It has been shown that inflammatory cytokines can promote progression to leukemia in vivo [186]. In myeloid malignant cells, innate immune signaling is often erroneously amplified, an effect mediated through the toll-like receptors (TLRs) that physiologically senses pathogen-associated molecular patterns (PAMPs) and damage-associated molecular patterns (DAMPs) and promotes an inflammatory response [187]. The activated TLR axis results in the secretion of several cytokines from leukemic cells that increases cell viability [188]. The mutational landscape of preleukemic states and AML (e.g., *DNMT3A*, *TET2*) may also make the HSCs vulnerable to inflammatory signals that promote leukemogenesis [189]. A very recent study in AML patients described unique inflammatory signatures that correlate with worse prognosis [190]. These were derived from single-cell level data and comprised of atypical B cells, a dysfunctional B cell subtype, an increase in CD8+ GZMK+, an elevation of regulatory T cells, and the concurrent decrease in T cell clonal expansion. The authors have also created an “inflammatory gene” score that correlates with survival outcomes in patients with AML.

However, it is important to note that the effects of inflammatory signaling are context dependent [188]. Based on the cellular molecular and chemical context, the activation of one inflammatory pathway may lead to malignant clonal expansion, the activation of an alternative pathway or secretion of a cytokine might lead to clonal suppression, and other pathways might be passengers in the disease course.

Interestingly, the abundance of main energy substrates in the context of obesity, namely glucose and FFA, leads to the overloading of intracellular energy provision pathways, an overproduction of NADH and FADH2 carrying electrons in the mitochondrial respiratory chain, and the production of reactive oxygen species during cellular respiration at rates exceeding the neutralizing capacity of cellular antioxidant mechanisms [102]. This imbalance leading to the accumulation of reactive oxygen species (ROS) is referred to as oxidative stress. Oxidative stress is an inherent feature of obesity and insulin resistance (IR) [191]. Oxidative stress is considered to play a major role in carcinogenesis by inducing base modification and DNA damage leading to mutations of proto-oncogenes and tumor suppressor genes [192]. The precise role of oxidative stress in leukemogenesis remains controversial, while additionally, the application of ROS to induce blast cell death has been considered in the treatment of leukemia [193].

### Dysregulation of Cytokines and Adipokines

The adipose tissue exhibits diverse endocrine functions, being a source of numerous hormonally active molecules, collectively referred to as adipokines and, more specifically, adipocytokines to denote pro-inflammatory cytokines originating from the adipose tissue [194, 195]. The secretory and circulating profiles of these molecules are subject to the distribution of adipose tissue of origin (visceral or subcutaneous) and substantial changes from the lean state to increasing obesity severity [196]. HSCs are the epicenter of a careful balance between quiescence, self-renewal, and differentiation within the healthy BM milieu [197]. In inflammatory states, many cytokines, including IL-1, IL-3, IL-6, tumor necrosis factor-a (TNF-a), and interferon (IFN) together with several growth factors such as M-CSF, G-CSF, and GM-CSF, drive the equilibrium from the steady state to emergency hematopoiesis [198]. The dysregulation of cytokine secretion is a hallmark of leukemia and preleukemic states [198–200]. Several studies have shown that IL-1, IL-3, IL-4, IL-5, IL-6, IL-8, IL-10, IL-12p70, IL-27, IL-35 as well as GM-CSF and stem cell factor (SCF) are elevated in AML patients compared to healthy controls [186, 201–204]. IL-1b can stimulate the generation of cytokines such GM-CSF and IL-6, acting as an autocrine growth factor for AML blasts [198, 205]. Besides, pro-inflammatory adipocytokines could directly promote leukemic cell survival and/or resistance to treatment; this may in turn harbor important implications for potential therapeutic approaches. Inhibition of IL-1 signaling constitutes a prominent example. An endogenous IL-1β repressor cytokine and likewise the monoclonal antibody canakinumab reduce leukemic cell proliferation in AML xenografts [206]. AML blasts overexpress IL-1 receptor accessory protein (IL-1RAP), an indispensable component of IL-1 receptor-related signaling. Chimeric antigen receptor T cells or monoclonal antibodies targeting IL-1RAP exhibit cytotoxic activity and inhibit the proliferation of AML cells, respectively [207, 208].

The presence of obesity also exerts a significant impact on the circulating profile of main adipokines. Increases of leptin, resistin, and visfatin as well as decreases of adiponectin/leptin ratio accompany the expansion and/or the dysfunction of adipose tissue, and have been associated with a multitude of adverse obesity-related outcomes [209, 210]. Interestingly, in an abundance of observational studies, corresponding changes of adipokine levels in relation to leukemia have been ascertained. Decreased adiponectin concentrations are observed in AML [211, 212], adult and childhood ALL [212, 213], in non-treated vs. treated CML [214], and CLL [215]. Leptin levels appear markedly increased in ALL [212] and decreased in AML [212, 216] and CLL [9]. Visfatin levels appear decreased in childhood acute leukemia and tend to normalize following HSC transplantation [217]. On the other hand, visfatin reduces AML blast proliferation, and its inhibition increases the sensitivity to chemotherapy, through the regulation of miR-IL-17 signaling via the PI3K/Akt pathway [218]. Resistin, a pro-inflammatory adipokine, is expressed in human AML and ALL cells [219], while its levels appear increased in childhood ALL [213].

Adiponectin, an anti-inflammatory adipokine, suppresses pro-inflammatory cytokine secretion by myeloid cells and T-lymphocytes and preserves HSC self-renewal and capacity to proliferate upon stimulation, while on the contrary, the absence of adiponectin receptor signaling may lead to sustained chronic cytokine-mediated HSC activation [220••], which in turn may promote the pre-leukemic state of clonal hematopoiesis [221]. AML cells express the leptin receptor whereby leptin binding increases the synthesis of pro-inflammatory cytokines such as IL-1β, IL-6, and TNF-α [216, 222], while it exerts proliferative and anti-apoptotic effects [223, 224]. Conversely, in childhood ALL, a reduction of the expression of leptin receptor is observed on the surface of blast cells compared with healthy bone marrow cells [225], while remission after treatment is associated with an increased expression on circulating mononuclear cell populations [226].

A recent study has found that fasting inhibits the development of ALL but not AML in mouse models [227]. The authors have shown that that the development and maintenance of ALL is dependent on the decreased expression of the leptin-receptor (LEPR). They observed that fasting can inhibit the development of ALL by increasing the expression of LEPR and its downstream signaling through the protein PR/SET domain 1 (PRDM1). LEPR expression levels were also associated with the prognosis of pediatric patients with pre-B-ALL.

Another recent study has evaluated the role of pre-conditioning leptin levels in 524 patients with various hematologic malignancies in patients undergoing HSC transplantation [228]. Low levels of leptin were found to be an independent risk factor for an increased relapse risk. However, this marker did not show any correlation with overall mortality or non-relapse mortality. The effect was consistent in an independent validation cohort.

Collectively, perturbations of adipocytokine physiology are observed both in obesity-related adipose tissue dysfunction and leukemic disease, constituting a candidate pathogenetic link between the two conditions. Intervention targeting adipo(cyto)kine receptors or related signaling pathways may thus serve as targets for anti-leukemic therapy.

### Bone Marrow Adiposity and Bone Marrow Microenvironment

#### Metabolic Characteristics of Bone Marrow Adipose Tissue and Leukemia

Bone marrow adipose tissue (BMAT) constitutes over 10% of total adipose tissue mass in lean individuals [229] and features distinct metabolic and secretory characteristics. Obesity, insulin resistance and dysglycemia are associated with BMAT expansion while the opposite effect is observed after treatment with metformin [230]. BMAT adipocytes are a source of adipokines such as leptin and adiponectin and likely exhibit a pattern of adipocytokine secretion which defers from that of visceral adipocytes; specifically, the mRNA levels of pro-inflammatory cytokines (TNFα, IL-1β) decrease in response to high-fat diet in mice, in contrast to their increase in peripheral adipose tissue [231]. Given their localization and proximity to HSCs, secretory signals deriving from BMAT, as well as their perturbations observed in obesity and dysmetabolism, may directly influence normal hematopoiesis and/or contribute to development of hematological disease, namely leukemias of myeloid origin [97]. BMAT expansion has been shown to negatively regulate normal hematopoiesis and is accompanied by a reduction of HSCs [232, 233]. BMAT expansion also promotes the pre-leukemic clonal hematopoiesis of HSCs harboring the DNMT3A mutations through IL-6 signaling [234]. Furthermore, existing evidence indicates that AML cells disrupt normal hematopoiesis by means of distorting BMAT function and impairing erythron-myeloid maturation, which is in turn restored after administration of PPARγ agonists [235]. Furthermore, it has been demonstrated that AML blasts induce a phosphorylation of hormone-sensitive lipase in BMAT and promote lipolysis, which in turn increases the abundance of FFAs and utilization by AML cells [236]. On the other hand, BMAT may hinder leukemic growth in T-ALL: injection of mice with human T-ALL blasts resulted in substantially lower infiltration of adipocyte-rich tail compared to thoracic vertebrae. Furthermore, blasts localized in the caudal compartment exhibited a different surface marker profile, lower proliferation rates, and suppressed metabolism which was however accompanied by the induction of resistance to vincristine [237]. These findings indicate that the dynamic interplay between BMAT and leukemic blasts likely results in differential effects on cell proliferation, metabolism, and chemotherapeutic resistance depending on cell origin.

In obesity, there is an abundance of nutrients that are stored in both the peripheral adipose tissue as well as in the bone marrow adipose niche. Τhe high concentrations of glucose, FFA, and AA could provide the energy supply for the proliferation and survival of the nearby leukemia cells [238]. Bone marrow provides the primary microenvironment for the development of leukemia. Mesenchymal stem cells from bone marrow biopsies of pediatric ALL patients have been found to highly express genes related to adipose tissue generation like CCAAT/enhancer-binding protein (CEBP) and PPARγ implying that the bone marrow is closely engaged with the adipose tissue [239].

In AML, leukemia cells have been shown to induce production of IGFBP1 from the adipose tissue to reduce insulin sensitivity and enhance their glucose uptake, favoring survival [137]. Furthermore, gut dysbiosis, lower serotonin, and incretin levels induced by the leukemic cells collectively inhibit insulin secretion; promoting thus cancer glucose uptake [137]. ALL cells display the Warburg effect where they prioritize glucose uptake that is dependent on GLUT1 receptor for their metabolic demands [240].

FFAs are an alternative source of energy for the proliferation and survival of leukemic cells [238]. In AML, adipocytes cultured together with blasts display upregulated expression of several enzymes involved in the metabolism and transport of fatty acids such as hormone-sensitive lipase, lipoprotein lipase, and fatty acid-binding protein-4 [236, 241]. In addition, leukemia cells can induce adipocytes to secrete FFAs that they can in turn use them in their advantage building elements of their cell membrane [242].

Aminoacids (AAs) are an essential metabolic source for all cells including blasts, and can be produced by adipocytes. ALL blasts do not express asparagine synthase which synthesizes the essential aminoacid asparagin, and are thus are susceptible to treatment to the drug L-asparaginase which inhibits asparagine synthase and further depletes this aminoacid rendering the blasts vulnerable [243]. Obesity can impair asparaginase efficacy in mice transplanted with ALL cells and without altering the plasma asparagine or glutamine levels [76]. The adipocytes residing in the bone marrow may contribute to therapeutic failure of L-asparaginase by supplying necessary AAs circumventing the deficiency of ALL cells [76].

#### The Role of Adipose-Derived Stem Cells

Adipose-derived stem cells (ASCs) are a kind of mesenchymal stem cells that may be detected in the vascular portion of the adipose tissue [244]. ASCs are a source of several molecules that are thought to promote tumor development such as IGF-1, transforming growth factor beta 1 (TGFβ1), vascular endothelial growth factor (VEGF), hepatocyte growth factor (HGF), and IL-8 [245]. In the setting of ALL, human ASCs support the growth of cancer cell lines when co-delivered to xenografts, in a dose-dependent manner [246]. However, the role of ASCs is context dependent as they can exert pro- or anti-tumorigenic effects depending on the microenvironment [247]. ASCs can negatively affect anti-tumor immunity as they can inhibit the proliferation of NK cells the differentiation of dendritic cells into B- and T-lymphocytes [248].

### Other Emerging Mechanisms

A number of other pathophysiological features, common between obesity and leukemic disease, constitute additional putative, albeit incompletely studied mechanisms which could pathogenetically link the two conditions.

The chronic low-grade inflammation that accompanies obesity and insulin resistance is associated with dysregulation of different functional aspects, which collectively result in a multidimensional immune dysfunction and, prominently, T-lymphocyte senescence [249–251]. This may lead to impaired immune surveillance and an increased propensity to malignancy, including leukemias. Besides, immune dysregulation is a cardinal feature of various types of leukemias [252–255], while effector T cell senescence may mediate resistance of AML cells to immunotherapy with checkpoint inhibitors [256]. Alterations of gut microbiota are featured in obesity and dysmetabolism as well as in various forms of leukemia, and their impact on the modulation of the immune system constitutes a field of active research in both conditions [257]. Perturbations in circadian clock gene expression have been implicated in the pathogenesis of obesity and insulin resistance [258–260] as well as in that of various leukemia types [261–263], although the role of circadian clock genes in the regulation of leukemogenesis has not been fully elucidated. It is still unclear whether these common pathogenetic features between obesity and leukemia are subject to therapeutic modulation.

## Preventive and Therapeutic Perspectives

### Preventive Measures (Diet, Bariatric Surgery, Physical Exercise)

The unequivocal epidemiological relationship between obesity and incidence of acute and chronic leukemias [31, 34, 43, 51, 74] as well as the numerous mechanisms linking the two conditions render the commonly implemented weight loss strategies of potential importance for the prevention of leukemia. Hypocaloric diets and increased physical activity constitute the mainstay of weight loss schemes, complemented by medical therapies and bariatric/metabolic surgery.

Data on the effects of caloric restriction-induced weight loss on leukemia risk are lacking, likely due to the limited feasibility of reduced weight maintenance in cohorts of adequate size in the long term. On the other hand, available evidence points towards an association between the qualitative composition of diet and leukemia risk. Based on the findings of a meta-analysis, increased maternal consumption of Mediterranean diet components, such as fruit, vegetables, legumes, and fish, has been associated with a lower risk of childhood leukemia, mainly ALL, while preconception folic acid and vitamin supplementation may also exert a protective effect. Consistent trends were observed on account of childhood dietary habits, together with a possible added risk by increased processed meat consumption [264]. A case-control study among pediatric patients aged 5–14 years has attributed a protective effect of milk and dairy consumption and a detrimental effect of added dietary lipids on ALL risk [265]. In contrast, adherence to a Western dietary pattern in adults has been associated with an increased CLL risk, independently of Rai stage [266]. Apart from the quantitative and qualitative dietary features, chrononutrition offers another dimension along which dietary interventions may impact on the manifestation and prognosis of various diseases, including malignancies. Although clinical evidence has been lacking to date, the implication of changes in clock gene expression in leukemias [263, 267] together with the dynamic impact of time restricted feeding on the pattern of gene expression in several tissues [268] may offer new perspectives for the prevention and management of leukemias.

Similarly to dietary interventions, there have been no observations on structured exercise programs to assess the effect of physical activity on leukemia risk. Nonetheless, evidence from observational studies indicates an inverse relationship between leisure-time physical activity and risk of myeloid leukemias, whereas no such association seems to exist with leukemias of lymphoid origin [269]. Accordingly, an adequate, compared with an insufficient, level of moderate-to-vigorous physical activity seems to be protective of the composite incidence of MDS and myeloid leukemias, although this observation is mainly driven by a reduction of MDS occurrence [270].

Besides, apart from prevention, a secondary intervention program implementing caloric restriction and increased physical activity to achieve a more than 20% caloric deficit significantly impacted ALL prognosis among individuals aged 10–21 years old, as indexed by significant reductions of minimal residual disease following chemotherapy compared with matched historical controls [271].

Obesity pharmacotherapy is a relatively newly developed field, precluding long-term observations on the effects of specific drug classes of leukemia occurrence. The beneficial effects of bariatric surgery, which constitutes the most effective currently available modality for prolonged weight loss and metabolic amelioration, on malignancy risk have been validated in long-term cohorts of operated patients. A reduction of incident total hematological malignancies was observed in participants of the Swedish obese subjects cohort [272]. However, the scarce evidence on leukemia-specific incidence is less compelling [273]. It should be noted that acquired copper deficiency which occasionally occurs as a complication of bariatric surgery [274, 275] is a secondary and potentially reversible cause of myelodysplastic bone marrow changes [276, 277], although the potential for malignant transformation of MDS which develops in the frame of copper deficiency is unclear.

### Biomarkers

Several molecules which are altered in obesity and are associated with obesity-related complications [9, 35, 103, 278–282] seem to concomitantly play a role in the pathogenesis of certain leukemia types and/or exhibit prognostic attributes. Apart from their systemic hormonal actions, the expansion of BMAT in obesity and related functional adipocyte changes [283] may be hypothesized to at least partially mediate these associations through the modulation of the bone marrow paracrine microenvironment.

Levels of adipose tissue-derived acute phase reactants such as CRP, TNFα, or IL-6 constitute a striking paradigm; a higher CRP-to-albumin ratio at diagnosis has been associated with shorter overall survival in transplant-ineligible elderly patients with AML [284] and shorter treatment-free and overall survival newly in newly diagnosed CLL [285]. IL-6 induces pediatric AML cell resistance to chemotherapy-induced apoptosis in vitro, and accordingly, bone marrow IL-6 concentrations are negatively associated with event-free survival in pediatric AML [286]. Circulating IL-6 is elevated in ALL and CML, while in the latter case, higher levels are observed during the blast crisis phase of transformation towards AML [287]. TNFα may promote AML progression through activation of the NF-κB pathway [288]. Higher TNFα levels are also encountered in ALL cases and normalize after induction chemotherapy; furthermore, an incomplete suppression TNFα is associated with incomplete remission after induction chemotherapy [289].

Among leukemia subtypes, perturbations of various adipokines have been observed, the most thoroughly studied of which are leptin and adiponectin. Adipocytes secrete leptin proportionally to bodily fat stores and hence its levels strongly reflect the degree of adiposity [290]. Contrary to normal promyelocytes, promyelocytes in AML may express the leptin receptor, and accordingly be prone to leptin signaling-induced proliferative and anti-apoptotic effects [291]. Elevations of circulating leptin have been occasionally [292] but not universally [212, 293] reported in AML, as well as ALL [212]. Leptin levels are also increased in CLL and CML [294, 295] while they tend to normalize after successful imatinib treatment in CML [295]. Lower adiponectin levels are associated with adverse features in obesity such as visceral adiposity, adipose tissue inflammation, and dysmetabolism [12, 296, 297]. Accordingly, lower adiponectin concentrations have been ascertained among patients with MDS compared with matched controls [103, 279, 281, 298]. Similar observations have been made for adult and childhood AML [211, 212] while in the former case, adiponectin levels inversely correlate with the cellular burden of AML as indexed by LDH concentration and bone barrow blast proportion [212]. Adiponectin levels may also be lower in newly diagnosed CML [215] or prospectively rise after initiation of TKI treatment [214]. Similar observations have been made for adult ALL [212], although the evidence regarding CLL and childhood ALL is less convincing [279]. Serum visfatin, an adipokine positively associated with an adverse metabolic profile in obesity [27, 108, 299], exerts proliferative effects and induces resistance to chemotherapy in AML cells in vitro [218]. On the other hand, visfatin levels are lower in pediatric AL patients than controls and rise to control levels after HSC transplantation [217]. Resistin, another adipose tissue-derived biomarker with positive associations with visceral adiposity and IR [299–301], has been found to be higher in newly diagnosed and relapsed pediatric ALL compared with controls [302].

MicroRNAs (also miRs) are small, non-coding RNA molecules which can modulate gene expression, with a potential role in the pathogenesis of malignant disease [303]. Certain adipose tissue-derived microRNAs are expressed in the adipose tissue and may concomitantly play a role in the pathogenesis of leukemias being also potential biomarkers. miR-125b is highly expressed in the white adipose tissue, particularly in obesity [304]. The overexpression of miR-125b in mouse model induces B- or T-acute lymphocyte leukemia [303], while in humans, the homolog Hsa-miR-125b-1 is implicated in the translocations associated with B-ALL or AML [305]. Increased levels of miR-486-5p, which is also upregulated in obesity, are encountered in CML [306] and may attenuate CML-progenitor cell sensitivity to tyrosine kinase inhibitor therapy [307]. MiRNA-221 and -222 are overexpressed in the adipose tissue in obesity [308, 309] and may also modulate the sensitivity of leukemic cells to treatment in ALL [310], CML [311], and CLL [312]. Circulating miR-142-3p levels, which positively correlate with BMI, waist-to-hip ratio, and IR indices [313], are downregulated in AML and are associated with drug resistance [314].

### Candidate Therapeutic Agents Targeting Oncogenic Pathways of Obesity and Insulin Resistance in Leukemia

Obesity and dysmetabolism-related perturbations in oncogenic pathways that play a role in leukemogenesis offer attractive prospects for the treatment of various leukemia types. In the crossroads of the two conditions, interventions aiming towards the loss of weight and metabolic amelioration could prove beneficial as preventive strategies. The increase of IR and reduction of insulin secretion induced by leukemic cells through various pathophysiological adaptive changes, including elevated expression of insulin-like growth factor binding protein 1 and suppression of incretin response, have been proposed as mechanisms promoting leukemic cell growth [137].

IGF-1 and insulin receptor expression has been ascertained in leukemic cells in AML [130, 315, 316], ALL [317, 318], CLL [113], and CML [319]. The activation of these receptors and the subsequent signal transduction through the PI3K-Akt-mTOR pathway play a central part in the leukemic cell growth and proliferation [320, 321]. This renders the drugs that target successive steps of these pathway potential candidates for leukemia treatment. Targeting of IGF1R signaling using pharmacological inhibitors (NT157/OSI-906), neutralizing antibodies, or Sorafenib induces anti-proliferative effects on ALL [317], AML [114], and CLL [113] cells in vitro, respectively. Idelalisib is a PI3Kδ inhibitor which is approved for the treatment of CLL, with demonstrated activity also against B ALL cells [322]. Furthermore, mTOR inhibitors Everolimus and Temsirolimus have shown promising results as adjunctive agents together with traditional drug therapy against leukemia in various settings in preclinical and early phase clinical trials [323–328].

Accordingly, repurposing of agents commonly used for the treatment of obesity-related metabolic disease offers useful perspectives for leukemia treatment as depicted in Table 4.

**Table 4:** Selected preclinical and clinical studies addressing repurposing of drugs commonly used for obesity, insulin resistance, or type 2 DM for the treatment of leukemias

Agent	Target	Study	Main findings
Metformin	AMPK activation and downstream inhibition of mTOR activity	Scotland et al. 2010 [329]	• Concentration-dependent decrease in oxygen consumption in AML cell lines in vitro • Apoptosis induction-dependent on cell line (MOLM14)
		Green et al. 2010 [330]	• Reduction of synthesis of oncogenic proteins in AML cells • Proliferation inhibition and reduced survival of AML cells ex vivo • Inhibition of human AML cell growth in mouse xenograft models in vivo
		Rosilio et al. 2013 [331]	• Proliferation inhibition induction of apoptosis in human T-ALL cells in vitro
		Valkana et al. 2013 [332]	• Metformin suppresses CML leukemic precursors and Ph+ ALL cells
		Martinez Marignac et al. 2013 [333]	• Metformin is cytotoxic against Dasatinib-sensitive CLL cells in vitro
		Bruno et al. 2015 [334]	• Exposure of CLL cells to metformin reduces expression of proteins associated with survival and proliferation • Metformin induces CLL cell apoptosis and inhibits of cell cycle entry after CD40-CD40L ligation stimulus
		Adekola et al. 2015 [335]	• Metformin sensitizes CLL cells to Ritonavir in vitro
		Tseng 2020 [336]	• Neutral effect of metformin treatment on leukemia incidence among 610,089 type 2 DM patients
Glitazones	PPAR-γ binding/activation	Sugimura et al. 1999 [337]	• Troglitazone reduces cell growth in human eosinophilic, myelomonocytic, and myelomonoblastic leukemia cell lines via induction of a p21 cyclin-dependent kinase inhibitor.
		Hirase et al. 1999 [338]	• Rosiglitazone and Troglitazone induce apoptosis and monocytic differentiation on a HL60 promyelocytic leukemia cell line.
		Konopleva et al. 2004 [339]	• Rosiglitazone and Troglitazone induce apoptosis and differentiation in leukemic cells in synergism with retinoid X receptor ligands
		Liu et al. 2005 [340]	• Troglitazone exerts anti-proliferative and apoptosis-inducing effects on human AML K562 and HL-60 cell lines, through upregulation of bax and downregulation of survivin and bcl-2 expression
		Takenokuchi et al. 2006 [341]	• Troglitazone dose-dependently inhibits cell growth and induces apoptosis human B-ALL cell lines with t(14;18) translocation.
		Saiki et al. 2006 [342]	• Pioglitazone dose-dependently inhibits colony formation in human leukemia cell lines (20–71%) and primary leukemia cells (1–25%) without considerably affecting healthy HSCs
		Prost et al. 2015 [343]	• Pioglitazone exhibits synergism with Imatinib on inhibition of CML cells in vitro. • Pioglitazone eradicates non-cycling, Imatinib-resistant CML stem cells in vitro. • Pioglitazone administration to Imatinib-treated CML patients in chronic residual disease results in complete molecular response prsisting up to 4.7 years after withdrawal.
Fibrates	PPAR-α binding/activation	Scatena et al. 1999 [344]	• Bezafibrate, gemfibrozil, and clofibric acid inhibit proliferation and induce differentiation of human AML cell lines in vitro.
		Liu et al. 2006 [345]	• TZD18, a dual PPAR-α/-γ agonist inhibits growth of Ph(+) B-ALL cell lines in vitro to a greater degree than pioglitazone.
Statins	HMG-CoA-reductase	Friedman et al. 2010 [346]	• Among 254 patients with CLL, statin therapy at the time of diagnosis did not affect overall and treatment-free survival, but was associated with reduced need for therapy in a subset of patient with short follow-up.
		Shanafelt et al. 2010 [347]	• Among 686 newly diagnosed CLL patients with Rai stage 0 disease, neither baseline statin therapy nor NSAIDs use had an impact on time to initial therapy, irrespective. • Among those treated with Rituximab-containing schemes, statin therapy had no effect on time to salvage therapy
		Podhorecka et al. 2010 [348]	• Simvastatin promotes CLL cell apoptosis via a reduction of BCL-2/BAX ratio, without affecting healthy lymphocytes • Simvastatin and fludarabine/cladribine exert synergistic cytotoxic effects on CLL cells
		Yavasoglu 2013 [349]	• Atorvastatin and rosiglitazone promote apoptosis of CLL lymphocytes in vitro*.*
		Chae 2014 [350]	• Concomitant intake of statin and aspirin was associated with longer progression-free and overall survival among 280 patients with refractory/relapsed CLL treated with Fludarabine, Cyclophosphamide, and Rituximab compared to each agent alone or no therapy.
		Chow et al. 2016 [351]	• Among 231 patients with CLL statin therapy was associated with a longer time to first treatment (57.5 vs. 36.0 months, *p* < 0.02) after excluding those with 17p deletion.
		Henslee 2018 [352]	• Fluvastatin and atorvastatin inhibit proliferation of natural killer leukemic cells and enhance the cytotoxic effects of chemotherapy. • The effects are likely mediated by inhibition of the mevalonate pathway downstream of HMG-CoA-reductase
		Gimenez et al. 2018 [353]	• Simvastatin decreases CLL cell survival and enhances the effectiveness of venetoclax and ibrutinib on an in silico model validated in vitro.
		Righolt et al. 2019 [354]	• In a case-control study (*n* = 1385 and 6841, respectively) the use of low-potency lipophilic statins (Fluvastatin/Lovastatin) was associated with a lower risk of CLL (OR vs. non-users 0.64, 95% CI 0.45–0.92)
		Jang et al. 2021 [355]	• Concomitant statin use increases the rate of deep molecular response in patients with CML under TKI therapy (55.8% vs. 41.0% at 5 years for *n* = 88 statin users vs. *n* = 320 non-users)
		Brånvall et al. 2021 [356]	• Among 3279 CLL patients, statin use at any time or follow-up (*n* = 753) was associated with lower disease-specific mortality.
Aspirin	Cyclooxygenase-1/-2	Bellosilo et al. 1998 [357]	• Aspirin and salicylate but not other NSAIDs induce a dose- and time-dependent apoptotic effect on CLL cells in vitro, through activation of the caspase pathway
		Weiss 2006 [358]	• In this case-control study of 169 adults with acute leukemia and 676 matched controls, aspirin used was associated with lower leukemia risk (aOR 0.84; 95% CI 0.59–1.21)
		Iglesias-Serret et al. 2010 [359]	• Aspirin induces apoptosis in various human leukemia cell lines in vitro by modulating the Mcl-1/Noxa balance.
		Ross 2012 [360]	• In this case-control study of 670 newly diagnosed myeloid leukemia cases (420 AML, 186 CML) and 701 controls, aspirin use was associated with lower risk of leukemia among women (OR 1.60; 95% CI 1.04–2.47) but not in men,
		Liang 2021 [361]	• Aspirin in combination with chidamide exert anti-proliferative effects on MDS-derived AML cells, likely through inhibition of the PI3K/Akt pathway.

*ALL* acute lymphoblastic leukemia, *AML* acute myeloid leukemia, *AMPK* AMP-activated protein kinase, *CLL* chronic lymphocytic leukemia, *CML* chronic myeloid leukemia, *DM* diabetes mellitus, *HSCs* hematopoietic stem cells, *MDS* myelodysplastic syndromes, *mTOR* mammalian target of rapamycin, *NSAIDs* non-steroidal anti-inflammatory drugs, *OR* odds ratio, *PI3K* phosphoinositide 3-kinase, *PPAR* peroxisome proliferator-activated receptor

Metformin is a first-line agent for the treatment of type 2 DM. Metformin exhibits a multifaceted mechanism of action, predominantly through the activation of AMP-activated protein kinase (AMPK) [362]. AMPK-dependent intracellular pathways seem to play a pivotal role in oncogenesis, including leukemogenesis [363]. Various in vitro studies have demonstrated the anti-leukemic cellular properties of metformin; however, corresponding clinical data are to date lacking (Table 4).

PPARs partake in many aspects of cellular proliferation, apoptosis, and differentiation. Fibrates and thiazolidinediones (glitazones) constitute two widely used medication classes in hypertriglyceridemia and type 2 DM, respectively. Fibrates exert their actions through selective agonism of PPAR-α and lower triglycerides while increasing HDL levels [364], both typical components of “diabetic dyslipidemia.” Glitazones activate PPAR-γ and are used as insulin sensitizers. Both medication classes have demonstrated interesting anti-leukemic properties in preclinical studies (Table 4).

Aspirin and statins are used for the risk modification in patients with high cardiovascular risk or with established vascular disease. Furthermore, both classes seem to possess interesting anti-leukemic properties (Table 4). Most statin-related observations have been made in CLL cell lines, whereby statins exhibit anti-proliferative effects and synergism with purine analogues in vitro. Potential clinical benefits have also been noted, despite some concerns regarding a presumed reduction of the anti-tumor effects of agents targeting CD20, due to the induction of conformational CD20 changes by statin therapy [365].

### The Challenges of the COVID-19 Pandemic

The COVID-19 pandemic brought about an unprecedented crisis affects virtually every aspect of clinical care. Patients with hematological malignancies were particularly affected, due to the complexity of the management of their illness necessitating either adherence to a strict therapeutic schedule or a chronic proximity to healthcare services at various levels [366]. Already early in the course of the pandemic, the presence of particularly active, hematological malignancies was recognized as a factor associated with frequent severe acute respiratory syndrome coronavirus 2 (SARS-CoV-2) acquisition and a more severe disease course [367]. Patients with leukemia are at a particularly high risk of COVID-19 due to factors associated with leukemic disease itself or its treatment (among others, leukopenia and lymphopenia, impaired cellular and humoral immunity, hypercoagulability, organ dysfunction) [368]. CLL patients treated with anti-CD20 agents (i.e., Rituximab) constitute a unique patient collective with regards to COVID-19; apart from the hypogammaglobulinemia associated with CLL, treatment-induced B-lymphocyte depletion further impairs the effective immunity development after receiving standard vaccination schemes [369], while it is also associated with impaired viral clearance in case of SARS-CoV-2 infection [370], occasionally with prolonged viral shedding [371]. Furthermore, blood product transfusions which are an inseparable component of leukemia management received a significant negative impact particularly in the early stages of the pandemic due to initial concerns regarding virus transmissibility as well as blood donation volume reductions and blood bank reserve depletions [366].

The necessity for timely therapy, including stem cell transplantation where indicated, following leukemia diagnosis and adherence to (often long term) treatment schemes should be weighed against the acute detrimental effects of treatment on the immune status of leukemia patients and subsequent risks of COVID-19 acquisition and adverse course, particularly around the peak of pandemic waves. Given the impact of adequate therapy on leukemia prognosis, there is little room for compromise with respect to delays or modification on treatment schedule. In peak pandemic periods, risk minimization strategies should be pursued [368], together with meticulous COVID-19 diagnostic screening even in asymptomatic individuals. Additionally, rationalization of transfusion strategies on a case-by-case basis is necessary in the face of blood product shortages. Lastly, although no uniformly accepted strategy exists for the passive immunization of leukemia patients against COVID-19, a more meticulous vaccination schedule could be chosen for selected patient groups, since repeat or multiple vaccinations have been shown to increase seroconversion rates in patients with impaired humoral immunity in the frame of B cell neoplastic disorders and their therapies [369, 372].

## Perspectives and Conclusions

Understanding the association between excess body weight and leukemia may present important implications for the prevention and treatment. Obesity represents an interesting risk factor for leukemia, being among the only known risk factors that could be prevented or modified while current research is mainly focused on the development of novel and expensive treatments for leukemia.

Emphasis on leukemia prevention could prevent several cases of leukemia. In the era of precision medicine, an important approach would be to perform large, multicentric, well-designed prospective studies to investigate whether obesity is a predisposing factor for the development of leukemia. As obesity is a modifiable factor, weight loss, healthy diet, and physical exercise may decrease the risk of cancers including leukemia [373••, 374–376]. Moreover, pharmacological interventions, repurposing drugs used for cardiometabolic comorbidities, and bariatric surgery may be highly recommended for leukemia and obesity-related cancer prevention [376–379].

Furthermore, the majority of studies evaluating the association between obesity and leukemia have used BMI as an index of obesity. Nonetheless, BMI presents several limitations when used as a marker of obesity, such as the lack of information regarding adipose distribution or visceral fat obesity [19, 63]. Other more reliable markers, such as waist circumference, waist-to-hip ratio, dual X-ray absorptiometry determinations, or magnetic resonance imaging, may be used. In terms of the pathogenetic mechanisms connecting obesity with leukemia, wider basic and translational research is required to further elucidate the complex molecular networks through which excess body weight influences the disease course providing potential therapeutic options.

Epidemiological evidence suggests a connection between obesity and leukemia. In addition, obesity is associated with worse outcomes and increased mortality in leukemic patients.

## Abbreviations

AAs: Aminoacids
AGA: Appropriate for gestational age
ALL: Acute lymphocytic leukemia
AML: Acute myeloid leukemia
AMPK: AMP-activated protein kinase
APL: Acute promyelocytic leukemia
ASCs: Adipose-derived stem cells
ATRA: ALL trans retinoic acid
BMAT: Bone marrow adipose tissue
BMI: Body mass index
BW: Birthweight
CCSS: Childhood Cancer Survivor Study
CEBP: CCAAT/enhancer-binding protein
CDC: Centers for Disease Control and Prevention
CEBP: CCAAT/enhancer-binding protein
CI: Confidence intervals
CLL: Chronic lymphocytic leukemia
CML: Chronic myeloid leukemia
CNS: Central nervous system
CRP: C-reactive protein
CRT: Cranial radiation therapy
CVD: Cardiovascular disease
DAMPs: Damage-associated molecular patterns
DM: Diabetes mellitus
DS: Differentiation syndrome
EFS: Event-free survival
ER: Estrogen receptor
EWAS: Epigenome-Wide Association Study
FFAs: Free fatty acids
GAL-9: Galectin-9
GH: Growth hormone
GWAS: Genome-Wide Association Study
HDL: High-density lipoprotein
HGF: Hepatocyte growth factor
HR: Hazards ratio
HSCs: Hematopoietic stem cells
IARC: International Agency for Research on Cancer
IFN: Interferon
IGF: Insulin-like growth factors
IGFBP: Insulin-like growth factor binding protein
IL: Interleukin
IL-1RAP: IL-1 receptor accessory protein
InsR: Insulin receptor
IR: Insulin resistance
IRS: Insulin receptor substrates
LDL: Low-density lipoprotein
LEPR: Leptin receptor
LGA: Large for gestational age
LIC: Leukemia initiating cells
MDS: Myelodysplastic syndromes
MiRs: MicroRNAs
mTOR: Mammalian target of rapamycin
NF-κB: Nuclear factor kappa-light-chain-enhancer of activated B cells
NSAIDs: Non-steroidal anti-inflammatory drugs
OR: Odds ratio
OS: Overall survival
PAMPs: Pathogen-associated molecular patterns
PFS: Progression-free survival
PI3K: Phosphoinositide 3-kinase
PPARα/γ: Peroxisome proliferator-activated receptor α or γ
RCT: Randomized controlled trials
ROS: Reactive oxygen species
RR: Relative risk
SARS-CoV-2: Severe acute respiratory syndrome coronavirus 2
SCF: Stem cell factor
SJLIFE: St. Jude Lifetime Cohort
S1P: Sphingosine-1-phosphate
TGFβ1: Transforming growth factor beta 1
TLR: Toll-like receptor
TNF-α: Tumor necrosis factor-alpha
VEGF: Vascular endothelial growth factor
vs: Versus
WHO: World Health Organization
y.o.: Years old
